# Arachnoid fibrosis in the cerebellopontine angle of primary trigeminal neuralgia: a histopathological study

**DOI:** 10.3389/fneur.2025.1536649

**Published:** 2025-04-11

**Authors:** Siqiang Tang, Mufang Huang, Jiajie Wu, Yexin Li, Kaiyuan Jiang, Peng Deng

**Affiliations:** The Central Hospital of Shaoyang, Shaoyang, China

**Keywords:** primary trigeminal neuralgia, microvascular decompression, arachnoid fibrosis, cerebellar pontine horn cisterna, Sirius red staining

## Abstract

**Objective:**

This study aimed to evaluate differences in the arachnoid membrane of the cerebellopontine angle (CPA) cistern between patients with trigeminal neuralgia (TN) and trauma patients without TN, providing novel insights into the pathogenesis of TN.

**Methods:**

Arachnoid specimens were collected from patients with primary TN undergoing their first microvascular decompression at the Neurosurgery Department of Shaoyang Central Hospital between January 2021 and September 2024 (study group) and from patients with posterior cranial fossa trauma undergoing surgery during the same period (normal control group). Specimens from both groups were subjected to hematoxylin–eosin (HE) staining and picric acid-Sirius red staining. Morphological thickness and collagen fiber thickness in the arachnoid membrane were measured under polarized light microscopy and then compared and statistically analyzed.

**Results:**

The study included 41 patients with primary TN and 38 normal control subjects. In the TN group, the mean thickness of the entire arachnoid layer in the CPA cistern was 87.86 ± 9.34 μm, and the mean thickness of collagen fibers was 53.95 ± 8.90 μm. In the control group, these values were 62.55 ± 1.55 μm and 33.50 ± 3.60 μm, respectively. The differences in both arachnoid thickness (*p* < 0.001) and collagen fiber thickness (*p* < 0.001) between the groups were statistically significant.

**Conclusion:**

Patients with TN exhibited significant arachnoid fibrosis and thickening in the CPA cistern, primarily due to an increase in collagen fibers. These findings suggested a potential pathological mechanism underlying TN.

## Introduction

Trigeminal neuralgia (TN) is the most common cranial nerve disorder encountered in clinical practice. It is characterized by severe, paroxysmal pain during attacks, significantly impairing the quality of life of patients. While the exact pathogenesis of TN remains unclear, several hypotheses have been proposed, including the central etiology theory, vascular compression theory (1, 2), viral infection theory, and arachnoid adhesion theory (3, 4). Among these, microvascular decompression (MVD) based on the vascular compression theory is currently considered the most effective treatment for TN (1).

Studies have reported arachnoid abnormalities around the trigeminal nerve root within the cerebellopontine angle (CPA) cistern, such as thickened arachnoid membranes, trabecular thickening, and adhesions. These changes can restrict, constrain, or even compress the trigeminal nerve root in some patients undergoing MVD (3, 5, 6). Abnormal CPA arachnoid changes, including thickening and adhesions, were also observed during MVD procedures. However, the underlying microstructural mechanisms causing these changes have not been reported in the literature, either nationally or internationally. Arachnoid fibrosis refers to an increase in fibrous connective tissue and a reduction in parenchymal cells, leading to tissue hardening and loss of elasticity. Continuous progression can result in structural destruction and dysfunction of the tissue. This raises the question of whether arachnoid fibrosis occurs in patients with TN.

In this study, we hypothesize that arachnoid fibrosis is present in the CPA arachnoid membrane of TN patients and may contribute to the pathogenesis of TN. By analyzing arachnoid specimens from TN patients, our study aims to elucidate whether arachnoid fibrosis is a contributing factor in TN pathogenesis. Understanding these pathological changes may provide novel insights into TN mechanisms and suggest new surgical strategies, such as optimizing intraoperative management of the arachnoid membrane, to improve long-term outcomes. Therefore, our findings have important clinical implications for refining TN treatment and reducing postoperative recurrence rates.

## Methods

### Clinical data

Trigeminal Neuralgia (TN) group: This group consisted of 41 patients diagnosed with primary TN who underwent MVD for the first time in the Neurosurgery Department of Shaoyang Central Hospital between January 2021 and September 2024.

The inclusion criteria for this study are as follows: (1) Participants must be diagnosed with trigeminal neuralgia, classified in accordance with the ICHD-3 criteria (7). (2) All patients with trigeminal neuralgia underwent prior treatments, including carbamazepine, radiofrequency ablation, and nerve blockade, but demonstrated inadequate pain control, prompting subsequent MVD.

**Figure 1:**
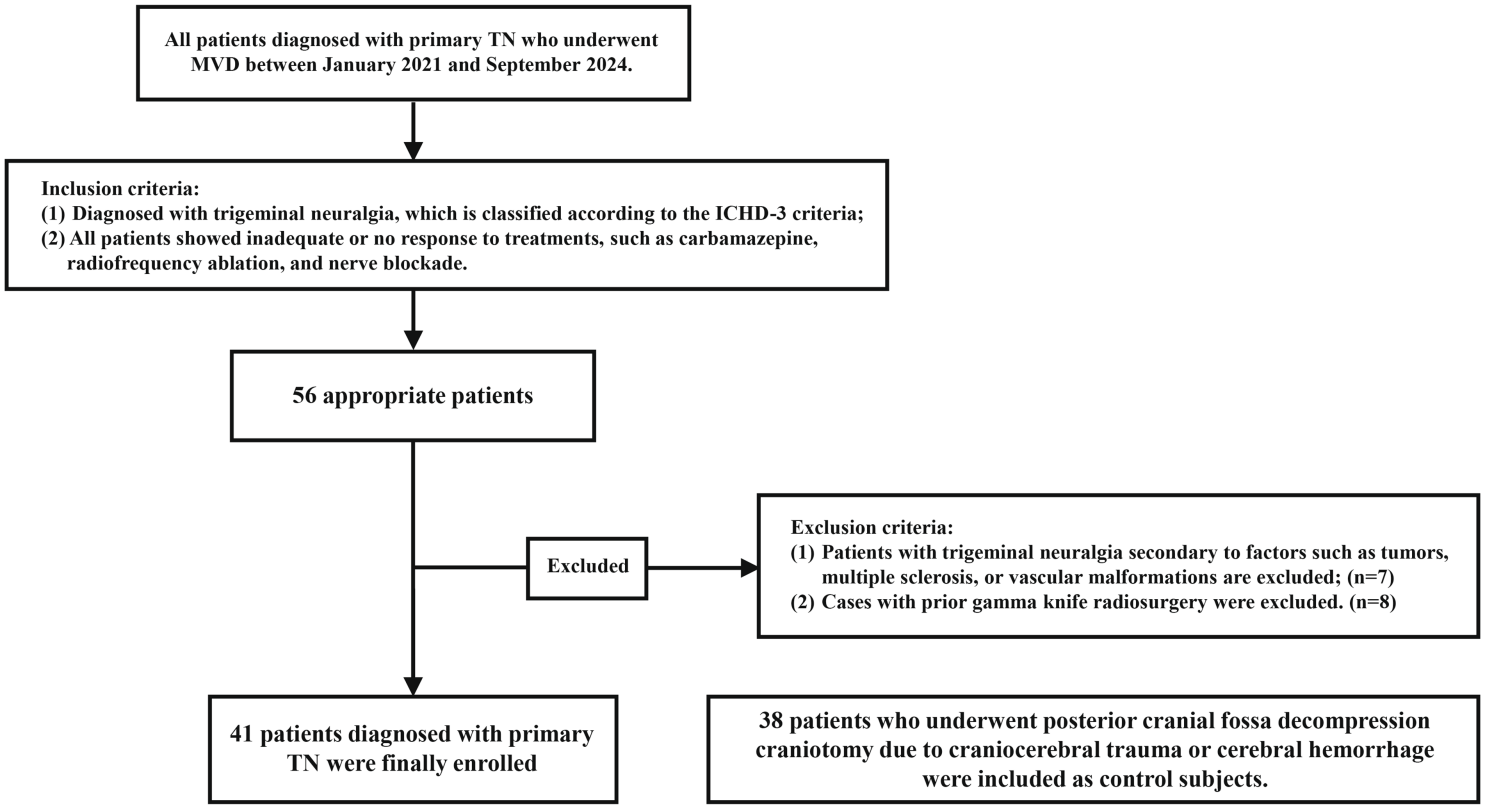
Flow chart of patient inclusion and exclusion. TN, trigeminal neuralgia; MVD, microvascular decompression.

The exclusion criteria are as follows: (1) Patients with trigeminal neuralgia secondary to conditions such as tumors, multiple sclerosis, or vascular malformations are excluded. (2) Cases with a history of gamma knife radiosurgery are also excluded. Normal Control group: This group comprised 38 patients who underwent posterior cranial fossa decompression craniotomy due to craniocerebral trauma or cerebral hemorrhage during the same period in the Neurosurgery Department of the same hospital. In the normal control group, patients with a history of intracranial tumors or trigeminal neuralgia were excluded. The detailed inclusion and exclusion criteria for both patient groups are outlined in Figure 1. Postoperative outcomes were evaluated one week after MVD. One week post-surgery, patients reported varying degrees of facial pain. According to the facial pain scoring standard of the Barrow Institute of Neuroscience (8),

### Specimen collection and retention

TN group: All patients underwent MVD using the retrosigmoid approach under general anesthesia. Routine craniotomy was performed with the patient in the lateral prone position, followed by microscopic entry into the CPA region. Normal control group: Following posterior fossa craniotomy, the outer arachnoid membrane in the CPA region was carefully collected to minimize contamination. All specimens were excised under a microscope and immediately fixed in 10% neutral formalin.

### Experimental methods

#### Preparation of paraffin specimens and staining

Both groups of specimens were prepared as paraffin sections and subjected to hematoxylin–eosin (HE) staining and Sirius Red staining staining. Briefly, the reagents for picric acid-Sirius red staining were prepared as follows. Sirius red (0.1 g) was mixed with 100 mL of a saturated aqueous solution of picric acid to prepare Sirius red-saturated picric acid solution. Hematoxylin (1 g) was dissolved in 100 mL of absolute alcohol (solution A). A solution of 30% ferric chloride (100 mL) was mixed with 1 mL of hydrochloric acid to prepare solution B. Solutions A and B were combined before use to prepare Weigert’s iron Hematoxylin staining solution. Moreover, a 0.5% solution of glacial acetic acid (100 mL) was prepared.

The staining was performed as follows. Paraffin sections were cut and deparaffinized in water, followed by staining with Weigert’s iron hematoxylin for 5–10 min. After rinsing three times with distilled water, the sections were stained with Sirius red-saturated picric acid solution for 20–30 min. Subsequently, the sections were immersed in a 0.5% glacial acetic acid solution for 2–5 s. Dehydration was then carried out directly using absolute ethanol, and the sections were cleared with xylene before being sealed with neutral gum.

#### Observation of arachnoid morphology and thickness

**Figure 2:**
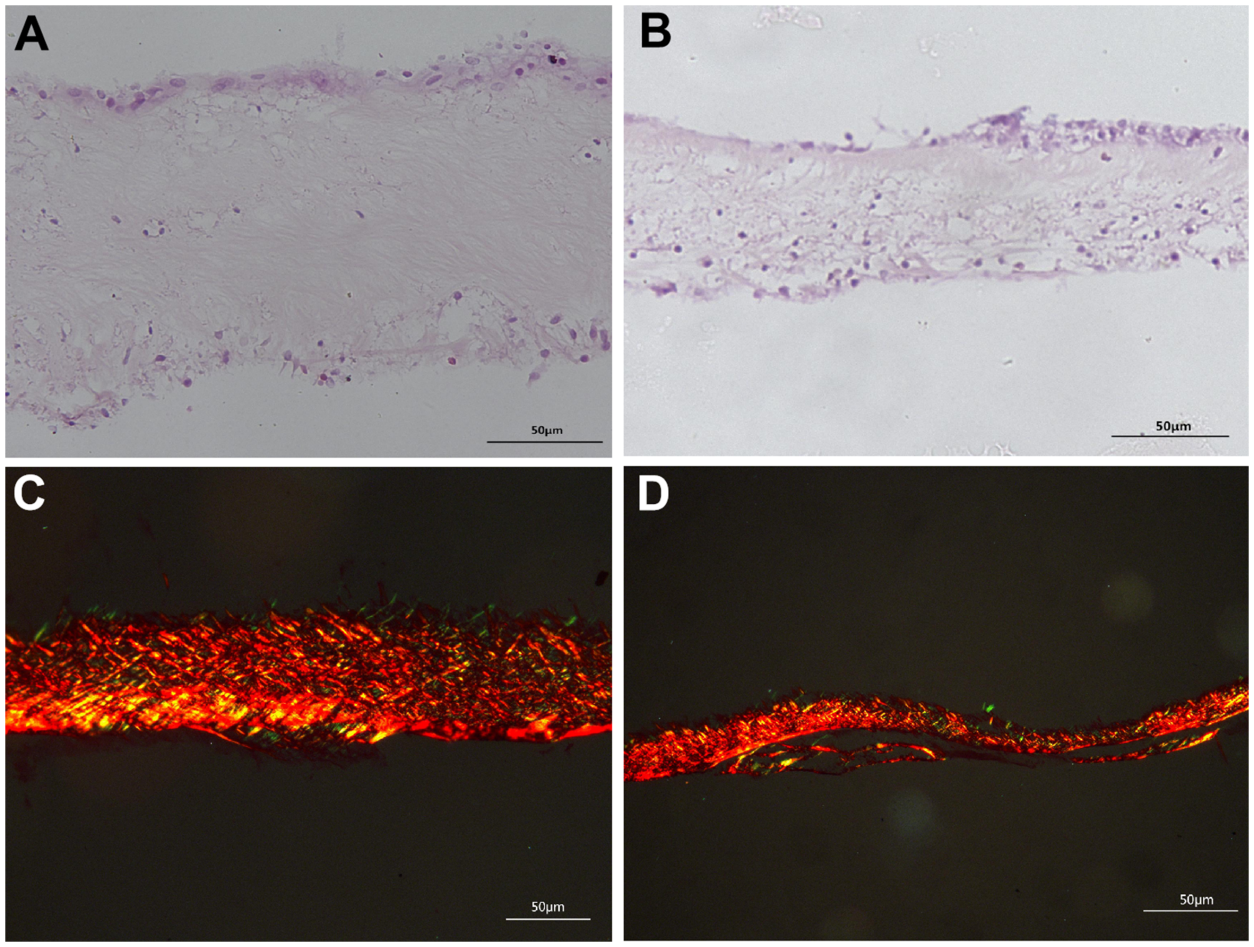
Histological analysis of the arachnoid membrane in patients with trigeminal neuralgia compared to normal controls, utilizing HE and Sirius red staining. The images demonstrate that the thickening of the arachnoid membrane in the trigeminal neuralgia group, compared to the normal control group, is primarily attributed to an increase in collagen fibers. **(A)** HE staining of the arachnoid membrane in trigeminal neuralgia patients, magnification ×400. **(B)** HE staining of the arachnoid membrane in the normal group, magnification ×400. **(C)** Polarized light imaging of collagen fibers in the arachnoid membrane of trigeminal neuralgia patients, stained with Sirius red staining, magnification ×400. **(D)** Polarized light imaging of collagen fibers in the normal group, stained with Sirius red staining, magnification ×400.

Two neurosurgeons observed the morphological structure of the arachnoid membrane under a Leica microscope using the HE-stained section specimens. The full thickness of the arachnoid layer in both groups was measured using the software ruler provided by the microscope. Figures 2A,B illustrate the microscopic morphology and thickness of the arachnoid membrane in the trigeminal neuralgia group and the normal control group, respectively. The final value was calculated as the average measurement obtained by both neurosurgeons.

#### Measurement of collagen fiber thickness

For the measurement of arachnoid collagen fiber thickness, the two neurosurgeons observed the morphology of collagen fibers under polarized light in all sections stained with picrate and Sirius red. The thickness of the collagen fibers in the arachnoid was measured using the software ruler on the microscope. Figures 2C,D show the arachnoid collagen fiber thickness in the TN and normal control groups, respectively. The final value was calculated as the average of the measurements taken by both neurosurgeons.

### Statistical analysis

All data were analyzed using the SPSS 24.0 software package. The Pearson Chi-Square test was employed to compare the age and gender between the TN group and the normal control group. The t-test was used to compare the thickness of the pia mater and the thickness of collagen fibers in the pia mater between the two groups. A one-way ANOVA S-N-K test was applied to compare the difference in pia mater thickness across different effect groups one week after surgery. A *p*-value of less than 0.05 was considered statistically significant.

## Results

### Clinical features

The TN group included a total of 41 patients, including 19 males and 22 females, with an age range of 32 to 68 years (average age of 55.14 years). The duration of the disease ranged from 9 months to 15 years, with an average duration of 3.7 years. Among patients with TN, 23 cases presented with left-sided pain, while 18 cases exhibited right-sided pain. Moreover, 32 patients had typical pain characteristics of TN, while 9 patients had atypical pain characteristics. A total of 12, 15, and 14 patients had a disease course of <3 years, 3 to 5 years, and > 5 years, respectively, with an average disease course of 3.7 years. During microvascular decompression surgery, the responsible vessel was found to be artery, vein, and combination of artery and vein in 29, 4, and 5 patients, respectively, while 3 patients had no responsible vessel. Observation under the surgical microscope showed that 24 patients exhibited obvious thickening and adhesion of the arachnoid membrane.
The normal control group included a total of 38 cases, including 20 males and 18 females, with an age range of 22 to 71 years (average age of 52.34 years). The normal control group comprised 38 subjects, including 15 patients with traumatic brain injury and 23 patients with cerebral hemorrhage

#### Postoperative effect at one week

Pain was completely resolved in 34 of the 41 patients, significantly relieved in 5 patients, mildly relieved in 2 patients, and none of the patients experienced ineffective results.

#### Comparison of clinical data and arachnoid membrane thickness between the two groups

**Figure 3:**
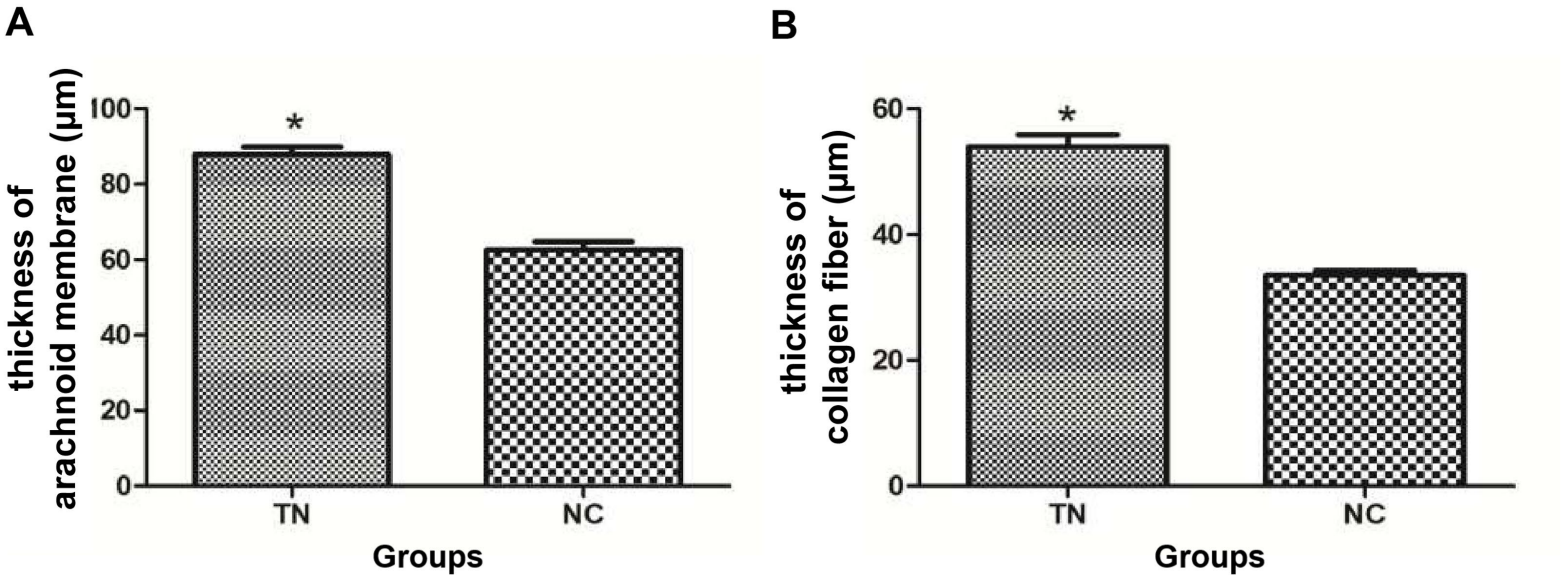
Statistical comparison of the thickness of arachnoid and collagen fibers between the two patient groups. **(A)** Comparison of full thickness between the normal and trigeminal neuralgia groups. **(B)** Comparison of thickness with collagen fiber of arachnoids between the normal and trigeminal neuralgia groups.

There was no statistically significant difference in age (*p* = 0.170) and gender (*p* = 0.576) between the TN and normal control groups. The thickness of the entire arachnoid layer in the TN group was 87.86 ± 9.34 μm, and that in the normal control group was 62.55 ± 1.55 μm. The thickness of the entire arachnoid layer was statistically different between the two groups (*P* <0.001, Figure 3A). The thickness of the collagen fibers in the TN group (53.95 ± 8.90 μm) was statistically significantly different from that in the normal control group (33.50 ± 3.60 μm) (*P* <0.001, Table 1 and Figure 3B).

**Table 1:** Comparison of data between the normal and trigeminal neuralgia groups.

Influencing factors	Trigeminal neuralgia group (*n* = 41)	Normal control group (*n* = 38)	*P*-value
Sex			0.576
Male	19 (46.3%)	20 (52.6%)	
Female	22 (53.7%)	18 (47.4%)	
Age (year)	55.14 ± 9.21	52.34 ± 8.73	0.170
Full thickness of arachnoid membrane (μm)	87.86 ± 9.34	62.55 ± 1.55	<0.001
Thickness of arachnoid collagen fibers (μm)	53.95 ± 8.90	33.50 ± 3.60	<0.001

#### Comparison of arachnoid thickness among different outcome groups at 1-week post-surgery

The complete arachnoid thickness in patients with TN was 86.14 ± 3.34 μm in the disappearance group, 90.60 ± 5.65 μm in the significant remission group, and 89.0 ± 2.78 μm in the remission group, with no significant differences among these groups (*p* = 0.343). Moreover, the collagen fiber thickness was 51.85 ± 5.23 μm, 49.2 ± 3.78 μm, and 52.50 ± 2.46 μm in the disappearance, significant remission, and remission groups, respectively, showing no significant difference (*p* = 0.365; see Table 2 for detailed information).

**Table 2:** Comparison in the arachnoid thickness, including whole floor and collagen fiber, among different outcome groups after microvascular decompression in one week.

Factors	Disappearance	Significant remission	Remission	*P-*value
Full thickness of the arachnoid membrane (μm)	86.14 ± 3.34	90.60 ± 5.65	89.00 ± 2.78	0.343
Thickness of arachnoid collagen fibers (μm)	51.85 ± 5.23	49.2 ± 3.78	52.50 ± 2.46	0.365

The *p*-value is the probability value of the overall difference in the ANOVA S-N-K test among the three outcome groups. Normality test *p* > 0.05.

## Discussion

Since the introduction of MVD surgery in clinical practice, the theory of vascular compression on the trigeminal nerve has gained increasing acceptance among neurosurgeons. However, several unresolved issues persist regarding this theory: why do normal individuals, upon undergoing magnetic resonance imaging (MRI) with thin-slice scans or autopsies, exhibit clear peripheral vascular compression on the trigeminal nerve root, yet do not develop TN? Conversely, in some MVD surgeries, even in the absence of obvious vascular compression, decompression surgery remains effective. Masatsune (9) suggested that this might be due to the relief of arachnoid constriction on the nerve root. According to this view, the adhesion and thickening of the arachnoid cause TN. The arachnoid trabeculae thickening and subsequent tension on the nerve root disrupt the synchronization of nerve root pulsation and cerebrospinal fluid fluctuation. This, in turn, increases the force exerted by the surrounding cerebrospinal fluid on the nerve root, potentially contributing to the development of TN. Similarly, Kim (10) *et al.* observed that during MVD surgeries, thorough dissection and loosening of the arachnoid around the nerve root and blood vessels are necessary to better expose and address the responsible vessels. Numerous studies (4, 6, 11–14) have documented the presence of arachnoid adhesion and thickening during MVD surgeries, especially in patients undergoing reoperation due to recurrence. However, these studies have not conducted in-depth pathological comparative investigations of the abnormal arachnoid.

The arachnoid is primarily composed of arachnoid cells and collagen fibers. Structurally, it can be divided into three layers: the arachnoid barrier cell layer, the basement membrane, and the loose arachnoid trabecula. The arachnoid cells, which are arranged in a palisade-like formation, exhibit regular and tight junctions in the layer adjacent to the dura mater, forming the arachnoid barrier cell layer. This highly cohesive layer restricts the passage of some macromolecules, thus acting as a selective barrier within the intracranial space (15). Beneath the barrier cell layer lies the basement membrane, predominantly composed of collagen fibers. These dense, resilient fibers contribute to the elasticity of the arachnoid. Picric acid-Sirius red staining is a specialized technique used to highlight collagen fibers. Sirius Red, a strongly acidic dye, binds to collagen fibers and exhibits birefringence properties under polarized light. When examined with a polarized light microscope, type I collagen fibers, which are tightly packed, display strong birefringence and appear as red or yellow fibers. In contrast, type II collagen fibers exhibit weaker birefringence and form loosely arranged networks of various colors. Type III collagen fibers show weak birefringence and appear as green, fine fibers, while type IV collagen fibers present a faint birefringence, with the basement membrane appearing light yellow (16, 17). This study represents the first use of Picric acid-Sirius red staining to conduct comparative analysis and measurement of the arachnoid within the CPA of patients with TN. The results reveal that the thickening of the arachnoid in TN patients is primarily attributed to the basement membrane, which is rich in collagen fibers. The accumulation of collagen fibers likely contributes to arachnoid thickening, restricting the surrounding blood vessels and nerves and potentially leading to arachnoid adhesions.

Compared to the normal control group, no significant changes were observed in the arachnoid barrier cell layer or the loose arachnoid trabeculae in patients with TN. However, a notable increase in collagen fiber content led to thickening and stiffening of the arachnoid membrane, resulting in a more fibrotic structure. This increased fibrosis caused a stronger adhesion of the arachnoid cuff to the nerve root. During MVD, the sharp separation of the arachnoid membrane proved more challenging than in standard craniotomies. Additionally, a closer anatomical relationship was observed between blood vessels and nerves, blood vessels and the arachnoid membrane, and the arachnoid membrane and the nerve root. To ensure complete separation of the offending blood vessels during MVD, it is essential to first perform a sharp dissection of the surrounding arachnoid membrane. Several studies (4, 6, 13, 18) have observed significant adhesion of the arachnoid membrane around the nerve roots in patients with recurrent TN who underwent repeat MVD surgery despite the absence of identifiable offending vessels. This strong adhesion of the arachnoid membrane tightly compresses the nerve roots against Teflon material, contributing to the recurrence of TN symptoms. The experimental findings of this study show an increase in collagen fiber content and thickening within the layers of the arachnoid mater in patients with TN, resulting in a more robust constraint on both the nerve roots and surrounding blood vessels. This creates a stronger restriction mechanism. Consequently, future MVD procedures should prioritize maximizing the separation of the abnormal segments of the trigeminal nerve root and associated blood vessels from adjacent meninges, ensuring complete decompression of both the nerve and blood vessels. Only thorough dissection and separation can significantly decrease the risk of re-compression caused by pressure from offending vessels (2).

The precise mechanism underlying the augmentation and thickening of arachnoid collagen fibers in patients with TN remains poorly understood. Collagen fibers, which are key components of the extracellular matrix, are secreted by arachnoid cells. In response to external stimuli such as inflammation, viral infections, immune factors, or abnormal hormone levels, these cells may increase the production of collagen fibers. Lhata (19) identified inflammation in the arachnoid web surrounding the trigeminal nerve root, suggesting that inflammatory processes within the arachnoid could stimulate enhanced collagen fiber secretion, leading to the thickening of the arachnoid layer. Furthermore, aberrant estrogen levels, commonly observed in TN patients (20), may also contribute to the excessive production of collagen fibers by arachnoid cells. In addition to these factors, genetic predispositions may play a role in congenital abnormalities in the content and thickness of arachnoid collagen fibers in TN patients, compared to those without the condition.

The thickness of the arachnoid matter does not have a significant impact on the short-term outcomes following microvascular decompression (MVD) surgery. During the MVD procedure, a thorough sharp dissection of the arachnoid was performed, allowing for adequate mobilization of the nerve roots and vessels, thereby alleviating the compression and constriction of the nerve roots by the arachnoid. This led to effective pain relief in patients postoperatively. However, over time, the thickness of the arachnoid may have a certain influence on the recurrence of pain in the long term, which necessitates further follow-up data to support this observation.

In conclusion, the accumulation of collagen fibers within the arachnoid in the CPA cistern of patients with TN leads to a thickening and stiffening of the arachnoid membrane. This structural change significantly impedes the trigeminal nerve root, promoting compression by surrounding blood vessels. The observed abnormal thickening of arachnoid collagen fibers in the CPA cistern offers novel evidence supporting the critical role of the arachnoid in the pathogenesis of TN.

However, this study has several limitations: (1) it is a single-center cross-sectional study with a relatively small sample size, necessitating further validation; (2) there may be measurement errors in the data; however, we took the average of measurements from two individuals to potentially reduce these errors; (3) other conditions, such as inflammation, may influence arachnoid thickness and fibrosis, possibly affecting the observed associations.

## Data Availability

The raw data supporting the conclusions of this article will be made available by the authors, without undue reservation.
